# Unusual mechanical failures of intrathecal baclofen pump systems: symptoms, signs, and trouble shooting

**DOI:** 10.1007/s00381-021-05154-3

**Published:** 2021-04-08

**Authors:** Janina Gburek-Augustat, Matthias Krause, Matthias Bernhard, Ina Sorge, Daniel Gräfe, Manuela Siekmeyer, Ulf Nestler, Andreas Merkenschlager

**Affiliations:** 1 Division of Neuropediatrics, Hospital for Children and Adolescents, University Hospital Leipzig, Leipzig, Germany; 2 Department of Neurosurgery, University Hospital Leipzig, Leipzig, Germany; 3 Pediatric Radiology, University Hospital Leipzig, Leipzig, Germany; 4 Pediatric Intensive Care Unit, Hospital for Children and Adolescents, University Hospital Leipzig, Leipzig, Germany

**Keywords:** Intrathecal baclofen therapy, Baclofen pump complication, Baclofen withdrawal, Baclofen overdose, Spasticity, Cerebral palsy, Bisphosphonate therapy

## Abstract

**Introduction:**

Although intrathecal baclofen (ITB) therapy is an effective treatment for spasticity, it has several disadvantages and a risk of complications.

**Methods:**

We present six pediatric patients who suffered from unusual mechanical failures of intrathecal baclofen pump systems.

**Results:**

With these case-vignettes, we provide a systematic approach on how to interpret the symptoms of ITB complications and an advice which further diagnostic and therapeutic steps to follow. We underline the seriousness of baclofen overdose, underdosing or withdrawal.

## Introduction

Reducing spasticity makes care easier, prevents secondary orthopedic problems and relieves pain. This improves the patient’s quality of life. Baclofen has an anti-spastic effect, but cerebral side effects often occur before therapeutic anti-spastic effects of oral administration are observed. To increase efficacy, intrathecal baclofen therapy (ITB) has been used to treat spasticity since the 1980s. ITB yields better reduction in spasticity at doses 1000 times lower than oral baclofen and minimizes adverse effects [1].

The indication for ITB treatment arises from the severity of spasticity and dystonia. Children with a gross motor function classification system (GMFCS) level IV or V benefit most. The treatment is palliative and constitutes a challenge with respect to the multimorbidity of the patients.

The baclofen pump is inserted subcutaneously in the lower abdomen and connected to a catheter as part of a surgical procedure. This catheter is placed under the skin and inserted into the intrathecal space at a lumbar level and then moved upwards. Placement of the tip of the intrathecal catheter has been suggested at the T1–T2 level for spastic quadriplegia, the T6–T10 level for spastic diplegia and in the midcervical region for dystonia [2]. The dose is adjusted individually, since in children there are no clear age-related or body weight-correlated recommendations [3]. Clinically, anti-spastic effects of continuous intrathecal dose changes occur with a 2–4-h delay [4]. A loss of efficacy in the long-term course in patients with a stable underlying disease (e.g. cerebral palsy) is seldomly observed [3].

Although ITB is an effective treatment of spasticity, it suffers from several disadvantages. The pump reservoir must be refilled with baclofen regularly through an injection. In addition, the pump must be surgically exchanged about every 5–7 years for battery exhaustion. Furthermore physicians must be aware of potentially serious complications (Table 1). A withdrawal syndrome is the most common cause of a life-threatening event, but toxicity from overdose due to mechanical system malfunction has also to be beared in mind [5]. In children, ITB complications occur in about 24–30% of cases [6–8], and the complications can present challenges for clinicians, so a high index of suspicion is needed to make the correct diagnosis [9].

**Table 1:** Symptoms and therapy of baclofen-underdosing/withdrawal and overdosing

	Underdosing/withdrawal	Overdosing
Mild symptoms	Return of baseline spasticity, pruritus, irritability, agitation, temperature > 38 °C, labile blood pressure, tachycardia, headache, disorientation, hallucination	Weakness (beginning in the lower limbs), tiredness to somnolence, listlessness, dizziness, constipation, urinary retention, nausea, vomiting, headache, drooling
Severe symptoms	Extreme CNS hyperexcitability, myoclonus, high fever (> 39 °C), altered mental status to coma, seizures, rhabdomyolysis, disseminated intravascular coagulation, multisystem organ failure, autonomic dysregulation to cardiac arrest, may advance to death when insufficiently treated	Muscular hypotonia to functional decline, hypothermia, bradycardia, arterial hypotonia, altered mental status to coma, seizures, increase of slow-wave activity and epileptiform discharges in electroencephalography, respiratory suppression to respiratory arrest, may advance to death when insufficiently treated
Differential diagnosis	Pain, anxiety, infection/sepsis/meningitis, epilepsy, malignant hyperthermia, intracranial haemorrhage, neuroleptic-malignant or serotonin syndrome	Hypoglycemia, electrolyte imbalance, epilepsy, infection /sepsis /meningitis, intracranial haemorrhage
Target of treatment	Restore intrathecal dose of baclofen used before underdosing as quick as possible	Remove residual ITB solution from the pump, lumbar puncture to remove CSF and reduce baclofen concentration
Bridging emergency treatment	Supportive care in an intensive care setting Baclofen p.o. (up to 150 mg/d) Benzodiazepine i.v. (1–2 mg/h continuously) Propofol i.v. Dantrolen i.v. (to treat rhabdomyolysis) Symptomatic treatment (intravenous fluids) Placement of an external lumbar catheter for administration of intrathecal baclofen	Supportive care in an intensive care setting Symptomatic treatment (e.g. respiratory support) No specific antidote available Seizure control according to guidelines (Physostigmine 0,02 mg/kg i.v. or i.m. can be considered)

## Patients and methods

We here describe six unusual mechanical ITB complications (Table 2), occurring in most of the patients after a long and successful treatment period, which obscured some of the hints to diagnosis and treatment of the failure (Tables 1 and 3). We use SynchroMed II pumps from Medtronic with corresponding Ascenda catheters. We applied a variable flow for patient case 6 and a fixed flow for all other patients.

**Table 2:** Overview of cases with mechanical ITB complication

Case	Localisation	Onset after pump implantation	Symptoms	Cause and findings
1	Pump	5 years	Sudden withdrawal symptoms.	Intraoperatively a corroded, defective pump was found (Fig. 1)
2	Connection from pump to catheter	5 years	Underdosing and indolent, nonreddened swelling in the abdominal area.	Connector from the catheter to the pump was not tight and baclofen and cerebrospinal fluid was leaking (Fig. 2)
3	Catheter	13 years	Underdosing	CT showed catheter rupture (Fig. 3)
	Catheter and pump		Pronounced calcification of pump, catheter and surrounding tissue	Calcification caused by treatment with bisphosphonates
4	Catheter	1 month	Underdosing	X-ray showed dislocation (Fig. 5)
	Catheter	4 years	Underdosing	X-ray fluoroscopy was assessed as inconspicuous, intraoperatively the catheter was found twisted (Fig. 6)
5	Epidural malpositioning of the catheter	From the beginning	Fluctuating symptoms: alternation between under- und overdosing	Spiral CT of the skull base and cranial spine after contrast medium application showed a faulty position of the tip of the tube system, which is placed epidurally (Fig. 7)
6	Intrathecal circulation problem	7 years after implantation of the pump-catheter-system and immediately after replacement	Fluctuating symptoms: alternation between under- und overdosing	Repositioning the catheter for high cervical baclofen delivery with high flow did improve the situation

**Table 3:** Step-by-step procedure for suspected baclofen pump problems

Clinical symptoms (see Table 1)							
Underdosing/withdrawal			Alternating underdosing and overdosing			Overdosing	
Possible causes							
Iatrogenic mistake: baclofen concentration too low or running rate too low	Mechanical problem of the pump	Problem with catheter	Mechanical problem of the pump	Malposition of intrathecal catheter tip	Problems with intrathecal baclofen distribution	Iatrogenic mistake: baclofen concentration too high or running rate too high	Mechanical problem of the pump
Trouble shooting/diagnostic steps							
Read out the pump							
Check the pump reservoir and refill the pump							
Laboratory tests: blood gas analysis, blood sugar, electrolytes, creatine kinase, creatinine, blood count, CRP, drug levels of anticonvulsants							
Programme bolus			X-ray of the pump	X-ray of the catheter	All other causes should be excluded:	-	X-ray of the pump
-	X-ray of the pump before and after the bolus	X-ray of the catheter		Empty catheter via side port and add contrast medium under fluoroscopy or CT-scan	X-ray of pump and catheter		
		Empty catheter via side port and add contrast medium under fluoroscopy or CT-scan			Empty catheter via side port and add contrast medium under fluoroscopy or CT-scan		
					External catheter in very high position with variation of flow		
	If still necessary, extended diagnostics can be considered: Radionuclide scintigraphy with serial sequential scanning after 24 h, 48 h, 72 h and/or MRI						
Problem-solving							
Problem solved by step “refilling”	Pump exchange	Catheter exchange	Pump exchange	Change position of the catheter	Increase baclofen flow and place catheter tip intrathecally in very high position	Problem solved by step “refilling”	Pump exchange

### Case 1

At the age of 11 years, the boy suffered from a traffic accident with considerable brain trauma. He developed a severe residual syndrome with spasticity GMFCS V and apallic syndrome. With 12 years a baclofen pump was implanted with good effect on muscle tone. The pump was refilled regularly.

Five years after baclofen pump implantation, for the first time a pump alarm arose. A few hours later, he presented at the emergency room because of high muscle tension, sweating and trembling. Creatine kinase was increased to 15.02 μkat/l (reference < 4.1). The suspected baclofen withdrawal symptoms were treated with oral baclofen, diazepam, continuous administration of analgesics and high-volume infusion therapy.

**Fig. 1:**
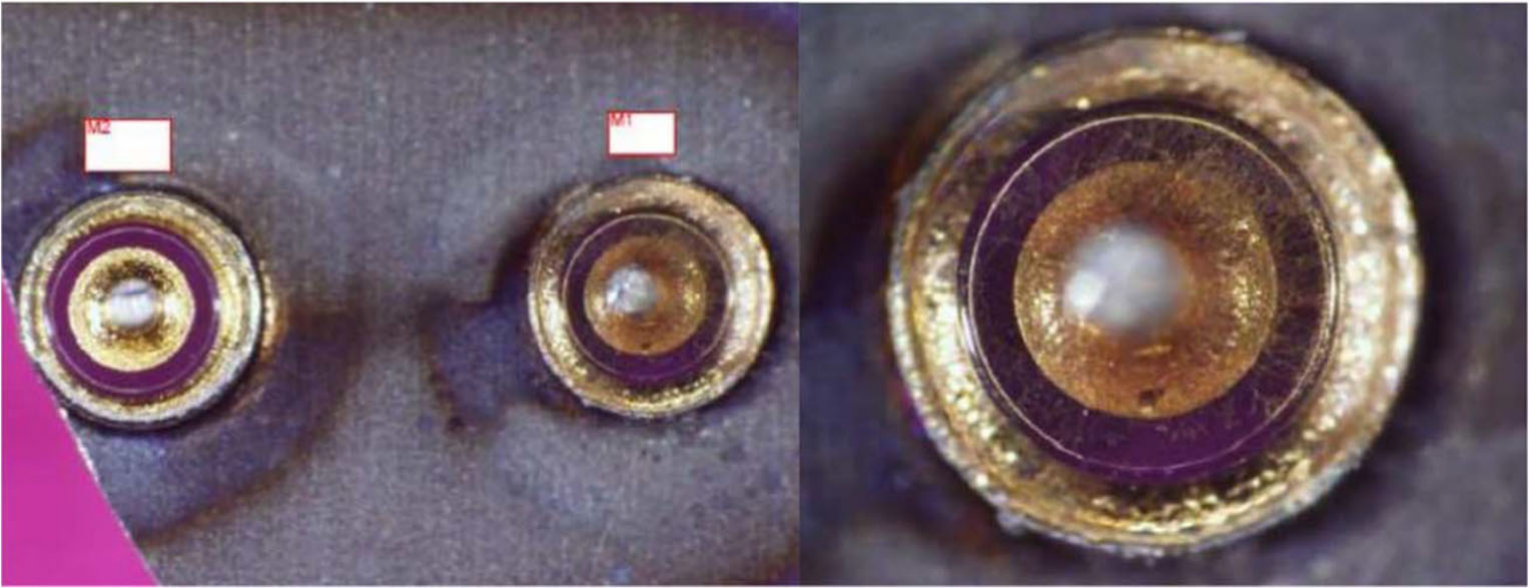
Case 1, photo of the corroded pump

In search for the cause, an X-ray was taken, and because this was unremarkable, contrast medium was applied via the side port of the baclofen pump to test the catheter system. No contrast medium flow into the catheter system could be shown. In a subsequent computed tomography (CT), no leakage was demonstrated, neither. Disconnection of the catheter was not detectable, so pump replacement was indicated. During intraoperative evaluation, a corroded and thus no longer functional pump was found (Fig. 1), a very rare complication [10, 11]. A new pump was implanted and the symptoms resolved.

### Case 2

**Fig. 2:**
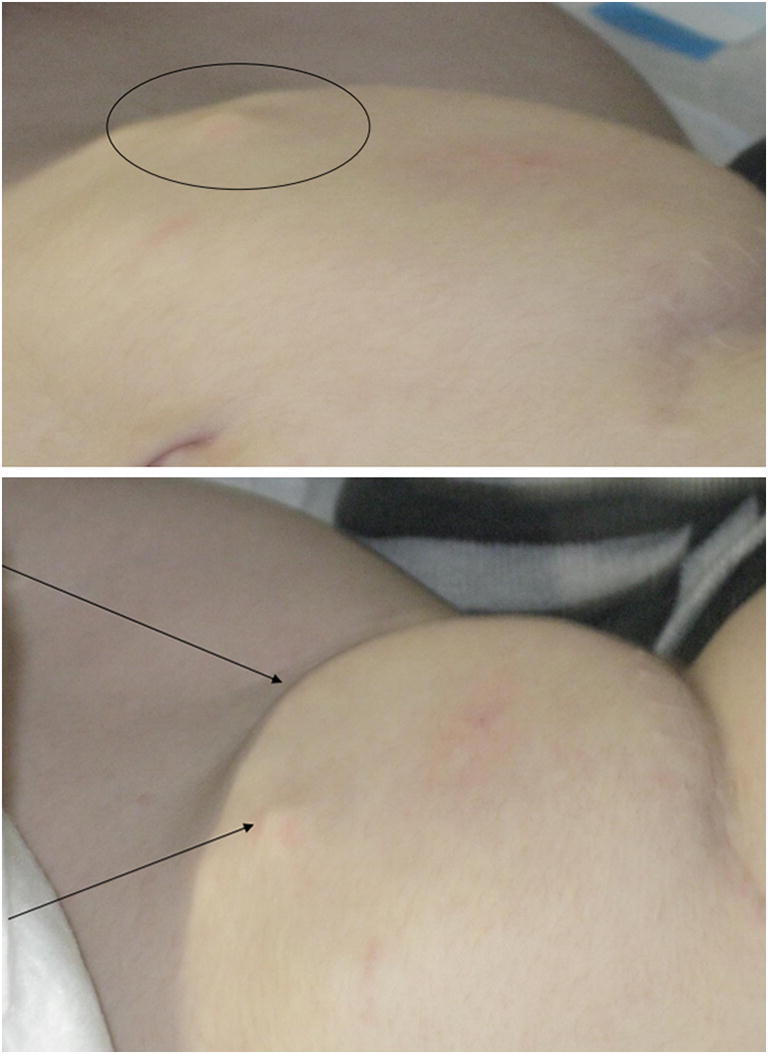
Case 2, photo of the abdomen shows subcutaneous bulging by dislocated catheter

The girl had fell victim to carbon monoxide poisoning at the age of 2 years. As a result, she developed spasticity GMFCS V and an apallic syndrome. A baclofen pump was implanted 1 month after the accident, and intrathecal baclofen therapy showed good effect on muscle tension. Five years later the patient was presented with a soft, indolent abdominal (Fig. 2). Spasticity had worsened in the previous weeks, so the baclofen dosage had been increased step by step. The swelling on the abdomen was punctured and serous fluid was collected. The fluid showed a high protein content (total protein 8.4 g/l) and no inflammatory cells, so it was interpreted as leakage out of the catheter. Surgical revision was performed, and the connector from the catheter to the pump was found to be loose. Baclofen was leaking from the proximal and cerebrospinal fluid from the distal part. The pump at the end of battery lifetime and the connector were replaced. Subsequently, a lower baclofen dosage was required to obtain sufficient treatment effects.

### Case 3

At the age of three, the boy suffered from hypoxic brain damage and subsequent bilateral spasticity GMFCS V as result of a drowning accident. Four months later, a baclofen pump was implanted with good effect. During the longer course, the patient developed inactivity osteoporosis and experienced several bone fractures. With 12 years, treatment with bisphosophonates was therefore initiated.

**Fig. 3:**
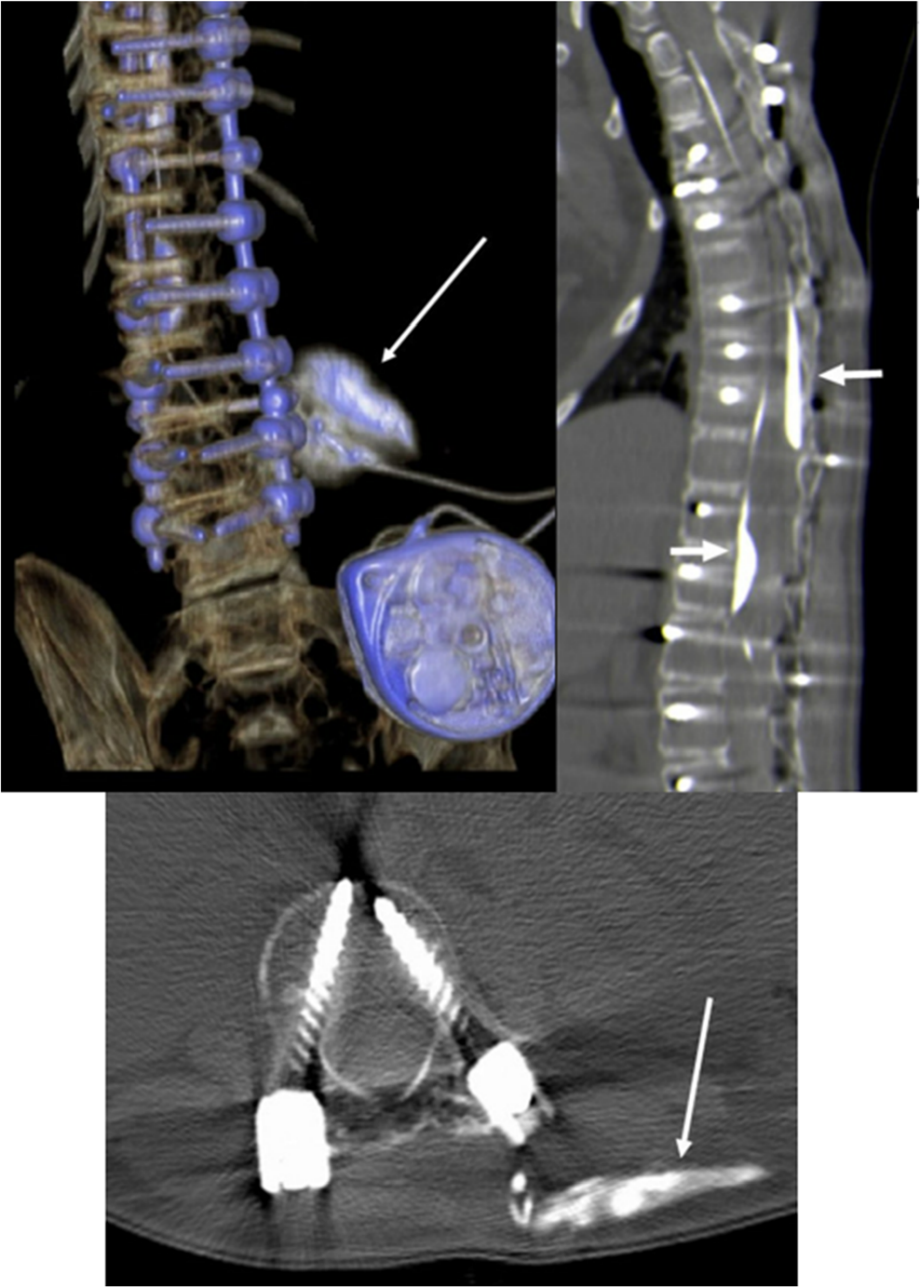
Case 3, CT shows catheter rupture: spiral CT of the spine after injection of the baclofen pump with 15 ml solutrast: evidence of a subcutaneous contrast medium extravasation as a sign of a tube rupture dorsal to L4 (long white arrow). In addition, most likely misalignment of the catheter tip with two subdural contrast agent deposits (short white arrows). Only minute amounts of contrast medium are displayed intrathecally

Overall, ITB was carried out in the patient for 13 years without any problems. Shortly before the planned explantation of the pump because of low battery charge, the patient’s spasticity worsened. A CT scan was performed under suspicion of catheter dysfunction which showed subdural contrast depots at two points and a subcutaneous catheter rupture (Fig. 3).

**Fig. 4:**
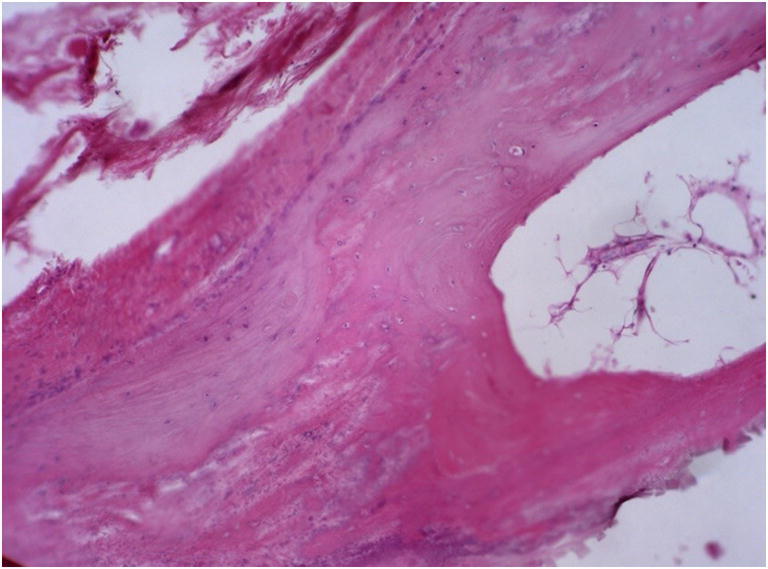
Case 3, histological workup displays steps of heterotopic bone formation with fibrous mesenchymal tissue lying adjacent to calcifying areas and immature woven bone (Scalebar corresponds to 100 μM)

For that reason, both pump and catheter were explanted. Intraoperatively, it was recognized that the tissue around the pump was hardly movable, calcareous coating of the pump was noticed, and the entry point of the catheter into the spinal canal was found to be surrounded by chalky, strongly adherent tissue. The calcification was very unusual and was interpreted as an adverse side effect of the bisphosphonate therapy (Fig. 4).

Six days after replacement, wound dehiscence developed with purulent secretion. The patient suffered from fever and laboratory results showed leukocytosis and increased serum CRP values. *Staphylococcus aureus* was identified in local wound specimen and blood culture. Antibiotic treatment with cefotaxime and clindamycin was started. The new pump and the entire catheter system had to be completely removed.

The patient then developed pronounced withdrawal symptoms after the pump explantation: shakiness, sweating, agitation and increase in spasticity. He received high doses of oral baclofen, midazolam, clonidine and dronabinol to alleviate the withdrawal symptoms. The patient’s condition slowly improved, and a new pump system was reimplanted several months later uneventfully.

### Case 4

Following peripartal varicella encephalitis associated with intracerebral haemorrhage, the newborn girl developed bilateral spastic cerebral palsy GMFCS V. At seven years ITB was started with an initially good response. The first complication occurred as soon as 1 month after pump implantation. An increase in spasticity and a higher frequency of crying periods were reported. By increasing the baclofen dose from 150 to 320 μg/d, no improvement of the symptoms could be achieved.

**Fig. 5:**
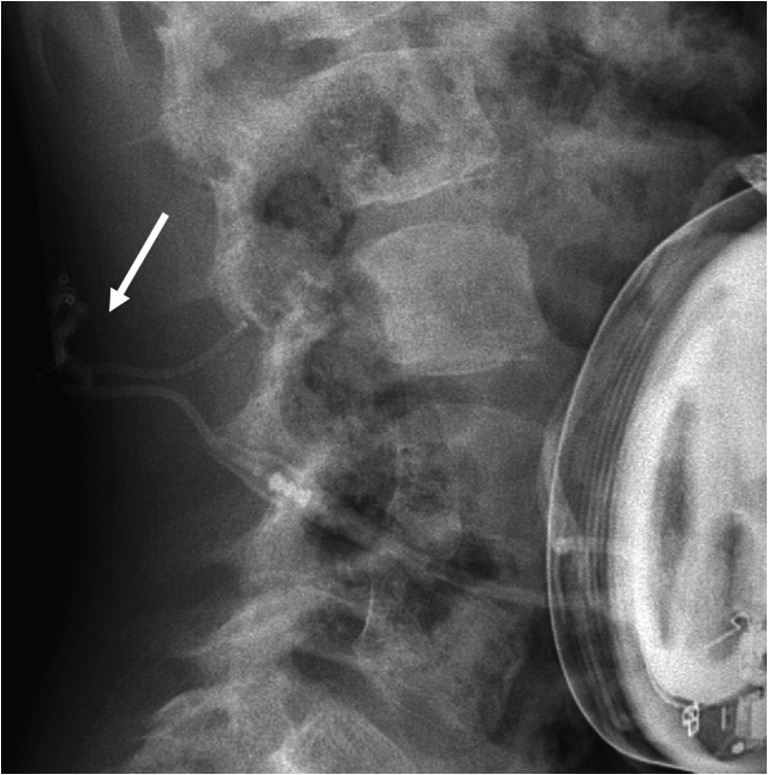
Case 4, lateral spine X-ray: the catheter is dislocated and a large part lies rolled up subcutaneously, so that it ends in the soft tissues between the lumbar spinous processes

X-ray demonstrated a dislocation of the intrathecal catheter (Fig. 5). The catheter was rolled up at the level of L3 and could be repositioned into the intrathecal space neurosurgically.

**Fig. 6:**
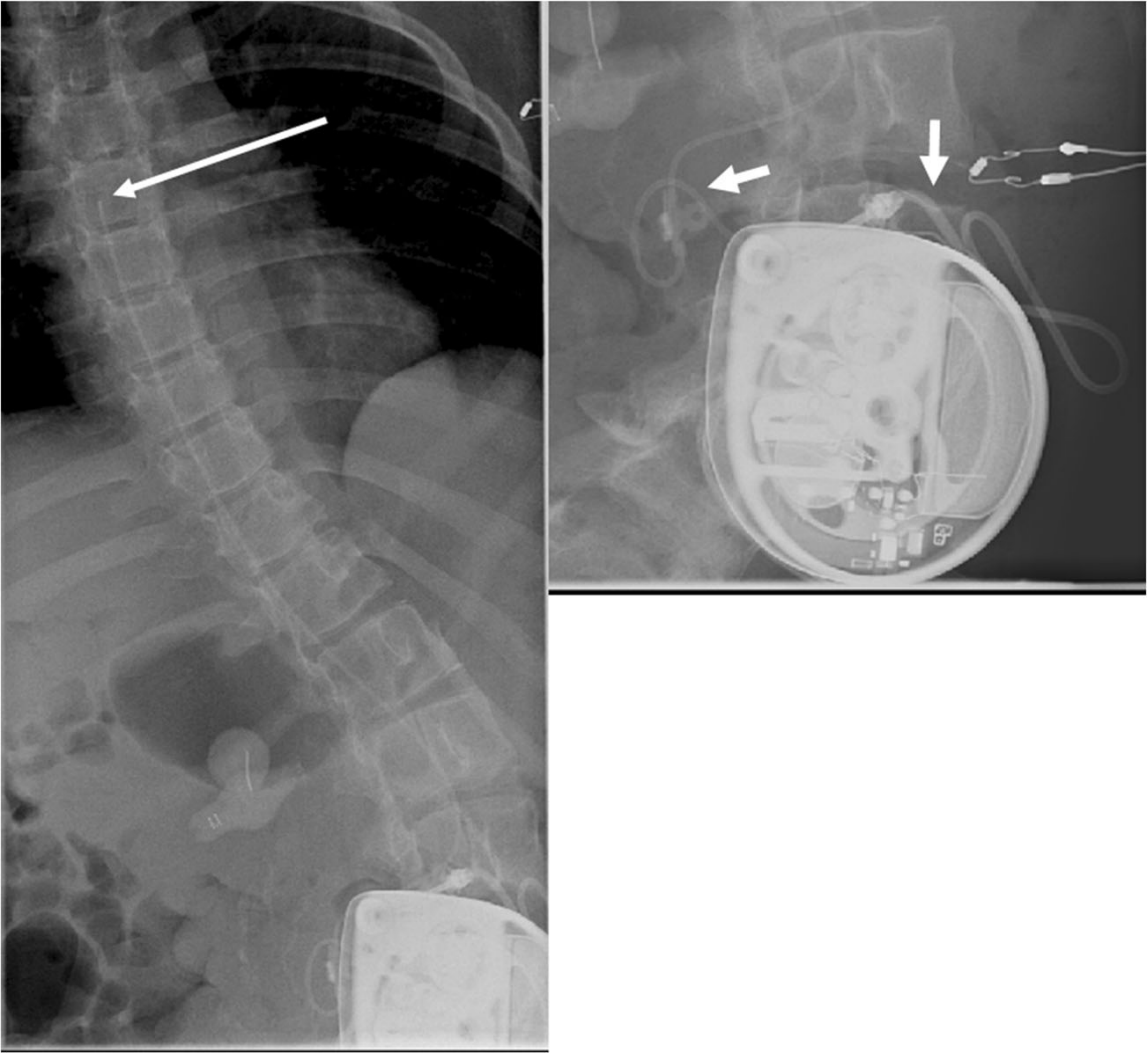
Case 4, X-ray fluoroscopy: after puncturing the access port of the pump, with correct needle position documented on the image, neither CSF can be aspirated nor contrast medium applied. The part of the tube system that is not covered by the pump is shown correctly, the tip of the catheter is projected at the level of the 6th thoracal vertebra (long white arrow). The short white arrows disclose the rolling up of the catheter

A long-term complication occurred 4 years later when spasticity and restlessness increased again. An X-ray was inconspicuous. Two months later, a discrepancy during pump filling was found. While 2.1 ml were supposed to be in the pump reservoir, 11 ml could be extracted. This showed a reduced extrusion of baclofen without programmed lowering of the flow rate. The catheter access port was punctured. Neither cerebrospinal fluid could be aspirated nor contrast agent injected. Surgical pump explantation revealed a coiled spinal catheter which had led to a functional closure of the catheter. Retrospectively, the detection of the once more subcutaneously twisted catheter on the X-ray images remains challenging (Fig. 6).

### Case 5

The patient presented with an Addison’s crisis and severe hypoglycemia as initial manifestation of X-linked adrenoleukodystrophy at the age of 4 years. He developed progressive leukodystrophy as typical feature of the underlying disease and bilateral basal ganglion necrosis which we interpreted as consequence of the severe Addison crisis. This resulted in a mixed picture of spastic and dystonic-dyskinetic movement disorder, initial GMFCS lay at III, decreasing to IV. One month after the neurological deterioration, baclofen pump was implanted.

**Fig. 7:**
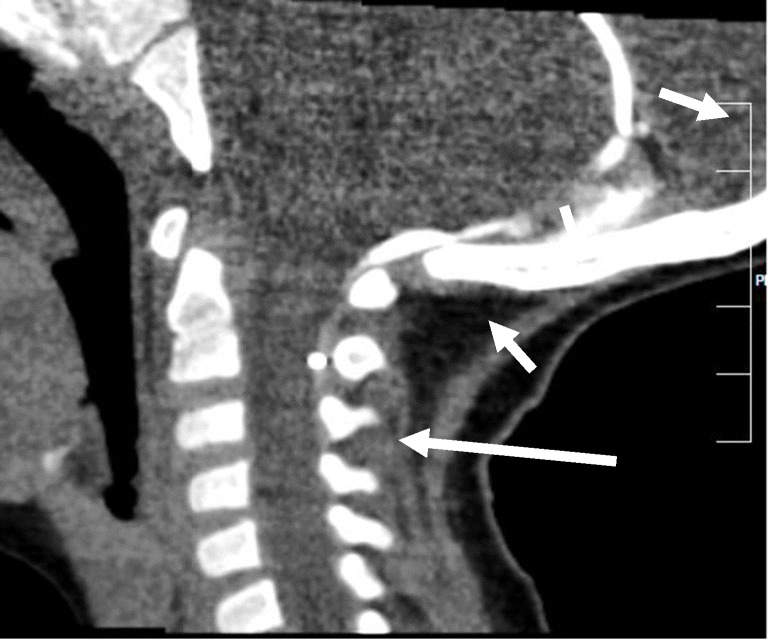
Case 5, spiral CT of the skull base and the upper cervical spine after contrast medium application via the side port of the baclofen pump: The tube system was intact up to the intraspinal entry of the tube at the level of L4/L5, without evidence of leakage. Contrast medium distribution presents epidurally dorsal to the CSF space at C2 (long arrow), pushing between the dural sheets of the tentorium (short arrow). There is no evidence of intrathecal contrast medium

Difficulties with the intrathecal baclofen therapy started nearly immediately. The symptoms varied widely with intermittent weakness in the legs. This was interpreted as a sign of overdose. These symptoms alternated with phases of increased spasticity suggesting underdosing. The fluctuating symptoms led to frequent reprogramming of the baclofen pump rate. The dose varied between 25 and 510 μg/d. CT was performed because of the unstable treatment results and revealed an epidural position of the dislocated intrathecal catheter (Fig. 7). This incorrect positioning most likely resulted in the delivery of changing amounts of intrathecal baclofen which explained the fluctuating symptoms. After revision of the catheter, baclofen was effective at a dose of 350 μg/d. During follow-up the dose had to be increased only as the disease progressed.

### Case 6

Periventricular leukomalacia because of preterm birth was the cause of bilateral spastic cerebral palsy GMFCS V in this patient. At seven years, the boy received a baclofen pump. ITB showed a good response until the baclofen pump was changed after 7 years when the battery was low. Three months after changing the pump, the patient developed a spastic crisis. X-ray was inconspicuous without signs of catheter interruption or leakage. A pump malfunction was assumed, and a replacement of the pump was planned. For short-term treatment of the withdrawal symptoms, an additional, external CSF catheter was placed to administer baclofen intrathecally which led to a rapid improvement. A new pump was implanted shortly afterwards and was connected to the remaining catheter system. However, during the following 5 months, problems persisted with increased intrathecal baclofen demand, interrupted by phases with signs of overdose as increased fatigue and somnolence. When analysing the difference between internal and external baclofen administration were twofold: the external catheter was positioned very high, and baclofen was more diluted leading doubled flow velocity. A rostral baclofen distribution problem has been discussed. For that reason a new spinal catheter was positioned to the level of C0/C1, and baclofen was thinned down to 1 mg/ml (compared to the standard dilution of 2 mg/ml) to allow for a reasonable intrathecal flow velocity delivered by the pump. This finally resulted in improvement and stabilisation of the previously unfavourable situation. Employing higher flow and extremely high catheter placement intraventricular placement could be avoided, what had been discussed as alternative solution.

## Discussion

Any change in the patient’s clinical state could be a warning sign of baclofen pump dysfunction.

Problems with ITB can be divided into three categories: first, there are application errors. These include incorrect programming or filling of the pump with the wrong drug dilution or failure to recognize a pending battery change. Second, there are mechanical flow problems. Here it is important to identify the localization: Is it the pump itself that has a defect and either delivers too much, too little or no baclofen at all, is it the connection point pump to abdominal catheter or abdominal catheter to spinal catheter part, is it in the course of the catheter or is it the intrathecal positioning of the catheter? Third, there are infections, here beyond the scope of this manuscript.

Depending on the dynamics of the onset and severity of the under- or overdosing symptoms, diagnostic and therapeutic steps should be initiated in a targeted manner. Symptom-related treatment should be initiated concomitant to the search for the underlying causes of malfunction (Tables 1 and 3).

An increase in spasticity may be a relative underdosing due to a therapeutic change in pump rate or because of an increased need with progressive worsening of the underlying disease. However, it is a warning sign, when spasticity suddenly deteriorates or when the dosage has been increased and the spasticity continues to worsen. At any level of the pump and catheter system, there might be the problem causing underdosing or withdrawal; therefore, each level must be checked step by step. Incorrect filling of the pump with low concentration of baclofen, lower dosage, a mechanical problem of the pump or failure to transport the drug out of the pump through the catheter into the intrathecal space due to dislocation, kinking, obstruction or malposition of the catheter tip, for example, can lead to a lack of baclofen at the site of action.

Overdosing occurs less frequently and may occur as result of an iatrogenic mistake when filling of the pump was incorrect (high baclofen concentration) or the pump rate too high. Another reason might be that the pump itself has a mechanical problem and is transporting more baclofen solution than programmed. Catheter problems mostly result in too little baclofen arriving intrathecally; therefore this usually does not lead to overdosing but eventually to fluctuating treatment responses.

If there are fluctuating symptoms with an alternation of over- or underdosing, this may be due to pump dysfunction. However, a malpositioning of the catheter tip, similar to case 5, should also be considered, when varying amounts of baclofen arrive intrathecally. Changes in intrathecal baclofen distribution may also lead to fluctuating symptoms, even though the catheter tip is correctly located intrathecally. We report this in case 6. Other causes of pump or catheter complications were made unlikely in this case by extensive diagnostic workup. Without proof of a mechanical dysfunction, improving the situation can be tried in these cases by placing the catheter tip to the C0/C1 level, as an alternative to intraventricular placement in case of efficacy decline.

Children who are immobilized by chronic disease are at high risk of developing secondary osteoporosis. The probability of receiving ITB and concomitant treatment with bisphosphonates is therefore increased. The intraoperative findings in case 3 with a combination of ITB and bisphosphonates with pronounced calcifications were impressive and so far not observed by us in other patients. We assume this to be a side effect of the bisphosphonates. We also suspect that these calcifications of the tissue contributed to the subsequent infection of the pump pocket. We could not find any other similar case report in the literature.

## Conclusion

We describe different causes of ITB failure, the resulting symptoms and signs, as well as an approach to trouble shooting.

Patients with spasticity GMFCS level IV and V may benefit significantly from ITB. However, the benefit should also be balanced against the risk of potentially life-threatening complications.

## Data Availability

Patient findings, imaging and records are documented electronically in the in-house hospital data system of the University Hospital Leipzig.

## Abbreviations

ITB: Intrathecal baclofen therapy
GMFCS: Gross motor function classification system
CT: Computed tomography
